# Sustainable catalysts for propylene synthesis

**DOI:** 10.1093/nsr/nwae273

**Published:** 2024-08-23

**Authors:** Mingyuan He

**Affiliations:** State Key Laboratory of Petroleum Molecular & Process Engineering, Shanghai Key Laboratory of Green Chemistry and Chemical Processes, School of Chemistry and Molecular Engineering, East China Normal University, China

The olefins industry is a cornerstone of the global chemical industry and plays a key role in the economy [1]. Propane dehydrogenation (PDH) is an on-purpose technology for propylene synthesis. Heterogeneous commercial catalysts containing Pt or CrO*x*, although widely used, are hampered by high cost, contamination or severe deactivation due to carbon deposition. Supported metal oxides have been shown to be active for C–H activation but have shown commercially unattractive performance [2]. Encouraged by the demands for environmental sustainability, both industry and academia are searching for environmentally friendly PDH catalysts related to the sustainable development of the olefin and chemical industries [3–9].

**Figure 1.**
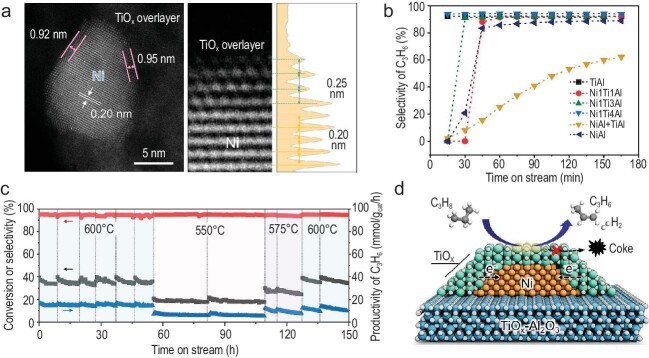
(a) *In situ* ETEM image of Ni@TiO*_x_* catalysts. (b) PDH performance over different catalysts. (c) Long-term test of PDH over Ni@TiO*_x_* catalysts. (d) Schematic of Ni@TiO*_x_* for PDH. Adapted and reprinted with permission from Ref. [10].

In the recent *Science* cover story ‘Defective TiO*_x_* overlayers catalyse propane dehydrogenation promoted by base metals’ [10], Gong and colleagues made a breakthrough in designing green and environmentally friendly catalysts for sustainable propylene synthesis. Titanium oxides, which are low-cost, naturally abundant and environmentally benign [11], and normally relatively poor catalysts for propane dehydrogenation, become highly active when fully encapsulated with metallic nickel. *In situ* environmental transmission electron microscopy (ETEM) provides visualized evidence of the evolution of TiO*_x_* overlayers on Ni nanoparticles (NPs) through the strong metal–support interaction (SMSI) at elevated temperatures (Fig. 1a) in agreement with spectroscopic observations, revealing the effective and precise modulation of the geometric and electronic properties of the catalysts.

The catalyst showed exceptional performance with 94% propylene selectivity at 40% propane conversion (Fig. 1b) and excellent stability during the long-term test (Fig. 1c). During successive dehydrogenation–regeneration cycles, reactivity could be recovered with little loss of Ni and Ti elements and without sintering or reconstruction. This catalytic performance surpasses most reported oxide-based catalysts and is comparable to that of noble Pt-based catalysts, while ensuring non-toxicity and low energy consumption during catalyst preparation and use. Gong and colleagues demonstrated the generality of this strategy by replacing either Ni with Cu or TiO*_x_* with ZnO. These efforts potentially address the need for environmental sustainability and the desire to reduce the carbon footprint of the chemical industry.

Combined experimental and theoretical techniques reveal that defective TiO*_x_* overlayers, composed of tetracoordinated Ti sites with oxygen vacancies (Ov), are catalytically active and their electronic states are modulated by electron transfer to subsurface metallic Ni. This changes the binding state of propyl and H intermediates, facilitating C–H bond activation and accelerating H_2_ desorption on TiO*_x_* overlayers, while subsurface metallic Ni participates only indirectly in the catalytic dehydrogenation cycle (Fig. 1d). The kinetic study also validated the changes in the active sites and the reaction mechanism upon formation of the Ni@TiO*_x_* structure.

This work illustrates a conceptual innovation in sustainable catalysis for propylene synthesis by combining conventionally less active titanium oxide with earth-abundant metallic nickel to form a defective TiO*_x_* overlayer encapsulated Ni@TiO*_x_*, paving the way for the next generation of efficient, cost-effective and sustainable propylene catalysts. This breakthrough is driving a technology revolution in the olefins industry, pushing the olefins and chemical industries towards greater efficiency, environmental friendliness and sustainability.
